# Comparison of real-time PCR and the Kato-Katz method for the diagnosis of soil-transmitted helminthiasis and assessment of cure in a randomized controlled trial

**DOI:** 10.1186/s12866-020-01963-9

**Published:** 2020-10-02

**Authors:** Beatrice Barda, Christian Schindler, Rahel Wampfler, Shaali Ame, Said M. Ali, Jennifer Keiser

**Affiliations:** 1 Swiss Tropical and Public Health Institute, Socinstrasse 57, 4002 Basel, Switzerland; 2 University of Basel, Basel, Switzerland; 3 Laboratory Division, Public Health Laboratory-Ivo de Carneri, Chake-Chake, Tanzania

**Keywords:** Soil-transmitted helminthiasis, Kato-Katz, PCR, Diagnosis

## Abstract

**Background:**

Diagnosis of soil-transmitted helminths (STHs) in developing countries is commonly based on microscopic detection of eggs in stool samples, using the Kato-Katz (KK) method, which has a poor sensitivity for detecting light intensity infections. We compared the performance of the KK method and real-time PCR in the framework of a randomized trial, which evaluated four novel treatments against *Trichuris trichiura* and concomitant STH infections.

**Results:**

Two stool samples obtained from 320 participants were examined at baseline and follow-up with quadruplicate KK and PCR analyses of one of the two samples using “bead-beating” for DNA extraction. At follow-up, 80 samples were negative according to both PCR and KK and 173 were positive with both methods for any of the STHs. Relative to PCR, the calculated sensitivity of KK at follow-up was 83.6%, 43.0% and 53.8% for *T. trichiura*, for hookworm and for *Ascaris lumbricoides*, respectively. The sensitivity of PCR compared with KK at this time point was 89.1% for *T. trichiura*, 72.7% for hookworm and 87.5% for *A. lumbricoides.* Cure rates (CRs) for *T. trichiura* and *A. lumbricoides* were slightly lower with the PCR method. For hookworm CRs with KK were mostly significantly lower, namely 36.7%, 91.1%, 72.2% and 77.8% for moxidectin, moxidectin in combination with tribendimidine, moxidectin in combination with albendazole and albendazole in combination with oxantel pamoate, respectively, whereas with PCR the CRs were 8.3%, 82.6%, 37.1% and 57.1%, respectively.

**Conclusions:**

In conclusion, a single real-time PCR is as sensitive as quadruplicate KK for *T. trichiura* and *A. lumbricoides* detection but more sensitive for hookworm*,* which has an influence on the estimated treatment efficacy. PCR method with DNA extraction using the “bead-beating protocol” should be further promoted in endemic areas and laboratories that can afford the needed equipment.

The study is registered at ISRCTN (no. 20398469).

## Background

The soil-transmitted helminths (STHs) *Trichuris trichiura, Ascaris lumbricoides* and the hookworms (*Ancylostoma duodenale* and *Necator americanus)* infect an estimated 1.5 billion people mostly in tropical and subtropical countries [1]. Chronic STH infections lead to malnutrition and malabsorption of macronutrients, growth impairment and decreased mental and cognitive development [2]. Children harbour most intense infections [3]. The WHO (World Health Organization) strategy to control these infections consists of large-scale distribution of albendazole or mebendazole (preventive chemotherapy) to pre-school and school-aged children once or twice a year according to the local prevalence, possibly complemented by implementation of hygiene and sanitation, and health education in the schools and communities [4].

Diagnosis of STH is usually based on microscopic detection of STH eggs in stool samples, with Kato-Katz being the most widely used method in human parasitology [5, 6]. However, the sensitivity of this method is highly dependent on the number of eggs excreted, the operator skills and the number of stool samples and slides analysed. Moreover, no quality control can be conducted in the diagnosis of hookworm eggs, since these are no more detectable after a couple of hours from sample-processing [7, 8].

In the past decades, alternative diagnostic options have been tested in order to increase the sensitivity and reproducibility of the diagnosis of intestinal helminths, most commonly molecular techniques [8–11]. Real-time polymerase chain reaction (PCR) is known to have a high sensitivity and specificity [12] and can be performed with a small quantity of sample. However, discordant results were observed when DNA extraction and PCR have been applied to helminth detection [9, 13]. Therefore, follow-up studies aimed to improve the DNA extraction method, which revealed to be the most cumbersome part of the process [7, 14–16]. Various techniques and commercial kits [9] following different protocols [7, 9, 14–17] have been tested. In contrast to viruses and bacteria, the wall of helminth eggs is difficult to lyse, therefore stress and longer lysing time are needed to improve the release of nucleic acids [11]. Protocols that include freeze–thaw cycles and physical stress result in a better performance compared to buffer methods [14, 18, 19]. Moreover, the “bead-beating” method, which adds a homogenization and freezing step with resistant beads offers a better disruption of ova and increases the sensitivity of the technique [7, 16, 20].

The aim of the present study was to evaluate real-time PCR as a powerful tool for helminth detection in a randomized controlled trial, which evaluated four novel treatments against *T. trichiura* and concomitant STH infection [21], in comparison to the performance of the widely used KK method. The important “bead-beating” process for DNA extraction of stool samples was established to a large throughput flow.

## Results

Table 1 shows the results for the selected 320 participants for KK and PCR at baseline and follow-up, respectively.

**Table 1:** Sensitivity calculated for 2 days of Kato-Katz (KK) and one PCR sample for *T. trichiura,* hookworms (incl. *A. duodenale, N. americanus* and *S. stercoralis) and A. lumbricoides*

	Sensitivity % (95% CI) Baseline	Sensitivity % (95% CI) Follow-up	Sensitivity % (95% CI) Total
***Trichuris trichiura***			
Combined KK	100	83.6 (77.6–88.5)	93.5 (91.3–95.7)
PCR vs combined KK	92.8 (89.4–95.4)	89.1 (83.6–93.2)	91.5 (89.0–93.9)
**Hookworm**			
Combined KK	70.4 (62.5–77.5)	43.0 (32.8–53.7)	60.0 (53.9–66.1)
PCR vs combined KK	77.5 (69.7–84.2)	72.7 (59.0–83.9)	76.2 (70.2.6–82.2)
***Ascaris lumbricoides***			
Combined KK	88.3 (82.8–92.5)	53.8 (25.1–80.8)	86.1 (81.3–90.9)
PCR vs combined KK	87.4 (81.8–91.7)	87.5 (47.3–99.7)	87.4 (82.7–92.0)

### Overall sensitivity of Kato-Katz and PCR

At baseline all samples were positive with KK for *T. trichiura,* according to the study design. At follow-up of all samples, 80 were negative according to both PCR and KK and 173 were positive with both methods for any of the three STHs.

Sensitivity of PCR and KK was calculated first for pooled baseline and follow-up samples. Relative to PCR, KK had a sensitivity of 93.5% for *T. trichiura,* 60.0% for hookworm and 86.1% for *A. lumbricoides.* Compared to the KK, PCR sensitivity was 91.5% for *T. trichiura*, 76.2% for hookworm and 87.4% for *A. lumbricoides* (Table 1)*.* Absolute numbers of the different types of concordant and discordant pairs for all three helminths and both time points with both diagnostic techniques are described in supplementary Table 1.

The PCR analyses allowed a differentiation of the two main hookworm species (*N. americanus* and *A. duodenale)* and *S. stercoralis* and out of 320 samples, only 1 harboured all three parasites, 4 were positive for both *A. duodenale* and *N. americanus*, 4 were positive for both *S. stercoralis* and *N. americanus* and 1 subject was infected with *A. duodenale.*

### Sensitivity at baseline

At baseline, inclusion criteria were positivity for *T. trichiura* with KK, thus the sensitivity of KK was 100% (Table 1). The sensitivity of KK was 70.4% for hookworm and 88.3% for *A. lumbricoides*. The calculated sensitivity of PCR vs KK was 92.8% for *T. trichiura*, 77.5% for hookworm and 87.4% for *A. lumbricoides* (Table 1)*.*

### Sensitivity at follow-up

Sensitivity for KK at follow-up was 83.6% for *T. trichiura,* 43.0% for hookworms and 53.8% for *A. lumbricoides* (Table 1). The sensitivity of PCR compared with KK was 89.1% for *T. trichiura*, 72.7% for hookworm and 87.5% for *A. lumbricoides* (Table 1)*.*

### Correlation between intensity of infection with Kato-Katz and PCR

In order to evaluate the agreement between the two techniques, we calculated the Spearman rank correlation taking into account only the positive samples. Results are shown in Table 2. The correlation was poor for hookworm (r = 0.35) at baseline and at follow-up (r = 0.27) and moderate for the other two parasites. The Cohen’s kappa (Table 3) between intensity categories based on combined KK EPG yielded a poor agreement (κ = 0.33) for *T. trichiura* at baseline, and a moderate one for the other parasites. At follow-up we observed a moderate agreement (κ = 0.47) for hookworm and a good one for the other two parasites (κ > 0.6).

**Table 2:** Spearman rank correlations between egg counts and copy numbers among samples which were positive according to both combined KK and PCR

	**R**	***p-value***	**n**
***Trichuris trichiura***			
Baseline	0.55	< 0.0001	297
Follow-up	0.56	< 0.0001	163
**Hookworm**			
Baseline	0.35	< 0.001	107
Follow-up	0.27	0.1	40
***Ascaris lumbricoides***			
Baseline	0.45	< 0.0001	166
Follow-up	0.39	0.38	7

**Table 3:** Agreement calculated with Cohen’s kappa between intensity categories based on EPG and frequency matched categories for PCR DNA copy numbers

	Kappa	*P* value	Agreement	Weighted Kappa
***Trichuris trichiura***				
Baseline	0.33	< 0.001	71.6%	0.35
Follow-up	0.61	< 0.001	79.4%	0.63
**Hookworm**				
Baseline	0.50	< 0.001	75.3%	0.50
Follow-up	0.47	< 0.001	85.0%	0.47
***Ascaris lumbricoides***				
Baseline	0.45	< 0.001	63.8%	0.57
Follow-up	0.74	< 0.001	98.8%	0.74

Intensity classes of infection calculated in eggs per gram (EPG) for: *T. trichiura* light (1–999); moderate 1,000–9,999, and heavy ≥ 10,000; for hookworm: light (1–1,999), moderate (2,000–3,999), and heavy (≥ 4,000); for *A. lumbricoides* light (1–4,999), moderate (5,000–49,999), and heavy infection ≥ 50,000

**Fig. 1:**
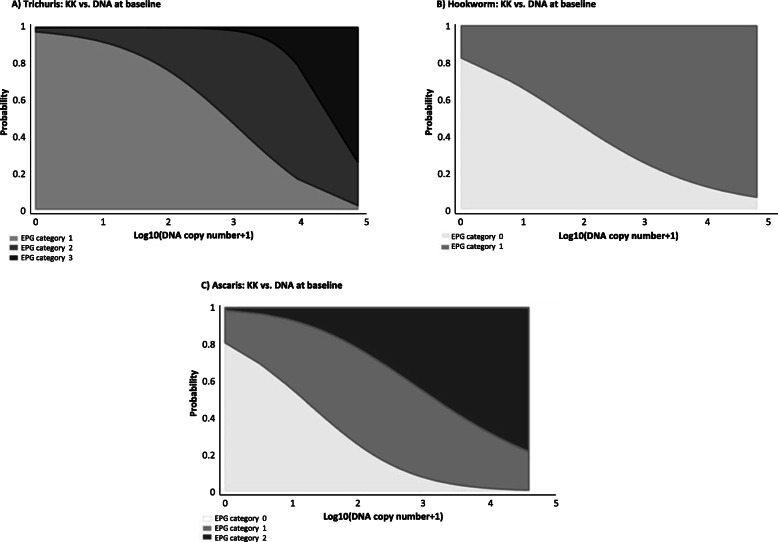
Probability of being in specific EPG-categories as a function of copy number. **a** *Trichuris trichiura* **b** Hookworm **c** Ascaris lumbricoides.* A: After excluding one extreme point with a copy number > 70′000

As shown in Fig. 1, based on the baseline data, high infection intensities (EPG counts) become more and more prevalent with increasing DNA copy number.

### Cure rates according to Kato-Katz and PCR

Efficacy (cure rates and egg reduction rates) of the four treatment arms based on KK were presented elsewhere [21]. In the framework of this study we compare cure rates obtained with PCR and KK for a subset of patients (Table 4). Although cure rates for *T. trichiura* and *A. lumbricoides* were slightly lower with the PCR method than with the KK method, these differences were not statistically significant. For hookworm, for two of the treatment arms, the calculated cure rates were significantly lower with PCR than with KK: 37.1 vs 72.2% (p = 0.004) for moxidectin-albendazole, and 8.3 vs 36.7% (p = 0.009) for moxidectin. For moxidectin-tribendimidine and albendazole-oxantel pamoate the difference between the respective cure rates, which were lower for PCR (82.6 vs 91.7% and 57.1 vs 77.8%, respectively) were not statistically significant (p = 0.24 and p=0.07, respectively). Odds ratios for different treatments compared to albendazole-oxantel pamoate (the reference regimen) for the three STHs according to KK and PCR are presented in Table 4.

**Table 4:** Cure rates according to two days of Kato-Katz (combined KK) and PCR for the four treatment arms

	Moxidectin	Moxidectin/ tribendimidine	Moxidectin/ albendazole	Albendazole/ oxantel pamoate
***Trichuris trichiura***				
Combined KK (95% CI)	15.0 (8.0–24.7)	24.7 (15.8–35.5)	50 (38.6–61.4)	82.3 (72.1–90.0)
OR combined KK (95% CI)	0.04 (0.02–0.09)	0.07 (0.03–0.15)	0.22 (0.1–0.44)	1.0
PCR (95% CI)	13.0 (6.1–23.3)	12.8 (6.3–22.3)	41.0 (30.0–52.7)	79.2 (68.0–87.8)
OR PCR (95% CI)	0.04 (0.02–0.1)	0.04 (0.02–0.09)	0.18 (0.09–0.38)	1.0
Difference	P = 0.73	P = 0.06	P = 0.26	P = 0.63
**Hookworm**				
KK (95% CI)	36.7 (19.9–56.1)	91.7 (77.5–98.2)	72.2 (54.8–85.8)	77.8 (60.8–89.9)
OR KK (95% CI)	0.17 (0.06–0.49)	3.14 (0.76–13.0)	0.74 (0.25–2.17)	1.0
PCR (95% CI)	8.3 (1.8–22.5)	82.6 (68.6–92.2)	37.1 (21.5–55.1)	57.1 (39.4–73.7)
OR PCR (95% CI)	0.07 (0.02–0.27)	3.56 (1.29–9.83)	0.44 (0.17–1.16)	1.0
Difference	P = 0.009	P = 0.24	P = 0.004	P = 0.07
***Ascaris lumbricoides***				
KK (95% CI)	97.7 (87.7–99.9)	97.9 (88.7–99.9)	94.1 (83.8–98.8)	95.9 (86.0–99.5)
OR KK (95% CI)	1.79 (0.16–20.4)	1.96 (0.17–22.3)	0.68 (0.11–4.26)	1.0
PCR (95% CI)	97.4 (86.2–99.9)	94.2 (84.1–98.8)	94.0 (83.5–98.7)	89.6 (77.3–96.5)
OR PCR (95%CI)	4.3 (0.48–38.5)	1.9 (0.43–8.42)	1.82 (0.41–8.08)	1.0
Difference	P = 0.93	P = 0.38	P = 0.98	P = 0.24

OR: odds ratio of cure rates calculated for the regimens with moxidectin, moxidectin/tribendimidine and moxidectin/albendazole, respectively, compared to the albendazole/oxantel pamoate regimen, for both Kato-Katz and PCR methods;

## Discussion

We compared the performance of real-time PCR and KK for STH diagnosis and its influence on drug efficacy in the framework of a randomized controlled trial investigating four novel broad-spectrum treatments against *T. trichiura* infection [21]. Real-time PCR has been widely applied for the diagnosis of helminth and other parasitic infections in the past few years and different protocols on DNA extraction have been tested [7, 15–17] but evaluations on the drug efficacy of novel treatments have not been conducted to date. In the current study, we assessed the sensitivity of PCR using the “bead-beating” method for the detection of STH on one stool sample compared to quadruplicate Kato-Katz analyses on 2 different stool samples.

We found that sensitivity of the two methods differs greatly between baseline and follow-up, which might be due to the inclusion criteria of the study, according to which only subjects positive (> 24 EPG) for *T. trichiura* by KK were enrolled. If baseline and follow-up results were pooled together, PCR is equal to KK for *T. trichiura* and *A. lumbricoides*, but more sensitive for hookworm. If we consider baseline and follow-up separately, at follow-up PCR is more sensitive than KK for all STHs. This finding is in line with the literature, with molecular diagnosis being more sensitive, even if not significantly different from microscopy [7, 15, 17].

So far there is no consensus, which parameter should be used to categorize intensity of infection by PCR: while for the moment some researchers continue to use Ct calculation when intensity is concerned [7, 15], we consider this parameter not reliable and reproducible, so we based our analyses on DNA copies. We therefore compared EPG and DNA copies, in order to find a pattern of predictability between the two diagnostic tests. Unfortunately, the Cohen’s kappa did not show any significance between measurements. We agree with others [15, 17, 22] that more studies are needed in order to find a better comparison parameter.

In the framework of our randomized controlled trial, we assessed the sensitivity of the two diagnostic methods in evaluating the efficacy of the different drugs used. The odds ratio was calculated for the three treatment arms (moxidectin, moxidectin in co-administration with tribendimidine and moxidectin in co-administration with albendazole) compared to the most efficacious treatment (albendazole in co-administration with oxantel pamoate). There was no significant difference between cure rates calculated for PCR and KK for *T. trichiura* and *A. lumbricoides*. However, for hookworm there was a considerable difference between the two methods, with PCR being significantly more sensitive than KK. Our findings are in slight contrast to a study by Vlaminck et al*.* who recently compared 5 diagnostic methods and tested the efficacy of albendazole against STH infections; based on their results, CR did not vary greatly if tested with different techniques [23]. Nonetheless, although the sensitivity of PCR was overall higher than the one of KK, also with this diagnostic method we confirmed that the treatment combinations tested had a good efficacy against STH infections.

Our result highlights that for diagnosing hookworm infections and assessing drug efficacy PCR outperforms the KK method. As already known, the accuracy of KK diagnosis for hookworm, especially at low intensity of infection, depends on several factors, such as irregular shedding of eggs by the worm, a non-homogenous distribution of eggs within the stool sample, degradation of eggs in the sample due to time till analysis and storage conditions [24–27]. PCR is also influenced by environmental or time conditions, since DNA can degrade in contact with DNAases, hydrolyse in acidic environment, or break by freeze–thaw cycles. However DNA of hookworms was shown to last for over 60 days mostly unaffected in 95% ethanol [28].

As well known, for all the studied parasites, PCR is burdened by the fact that it is still not clear what has been replicated during the process: the method, in fact, is not able to distinguish patent from pre-patent infections and does not differentiate between larvae, adults and eggs of the parasite [29, 30]. Some promising developments are showing that the detection of multicopy genes such as tandem repeats might overcome the challenge of DNA extraction of stochastic distributed eggs in stool samples [31, 32]. However, the poor correlation with microscopy, the differentiation challenge between dead and active infections as well as the interpretation of burden of disease will remain.

Interestingly, we have observed that almost all infections with hookworm were positive for *N. americanus* and only few were positive for *A. duodenale*; this is a relevant information for the distribution, epidemiology and treatment of the parasite. Moreover, as previously reported, *S. stercoralis* prevalence on Pemba island has dramatically dropped since the MDA with ivermectin for lymphatic filariasis. Our data support the evidence that *S. stercoralis’s* prevalence is nearly 0% on the island [33, 34].

Our study suffers from some limitations. As already mentioned, to be eligible for our study, subjects had to be positive for *T. trichiura* at least on one day of KK examination, which includes a bias for our diagnostic comparison. However, the follow-up samples allowed performing the sensitivity analyses. Furthermore, we only had a few high intensities of infections for all STHs both at baseline and follow-up and only one out of the two samples were collected for the PCR method, which impairs a thorough comparison between techniques.

## Conclusions

Our results show that a single PCR, based on the bead-beating extraction method, is as sensitive as quadruplicate KK for *T. trichiura* and *A. lumbricoides* detection but more sensitive than quadruplicate KK for hookworm, which is particularly evident after treatment*.* As we have demonstrated this has a strong influence on the calculation of hookworm cure rates.

We conclude that PCR should be promoted for STH detection in clinical trials and in laboratories that can afford the equipment needed. Further steps should be taken in order to clarify the correlation between DNA copies and intensity of infection detected with microscopic direct methods. We are also looking forward to newly developed molecular assays based on the detection of multicopy genes to overcome the challenge of stochastic distribution of eggs in stool samples.

## Methods

### Study design and laboratory procedures

Stool samples were collected in the framework of a phase II, randomized, single-blind clinical trial evaluating the safety and efficacy of moxidectin alone and in co-administration with albendazole and tribendimidine *versus* albendazole-oxantel pamoate co-administration against *T. trichiura* infection [21]. The trial was performed between April and August 2017 on Pemba Island, United Republic of Tanzania in school-aged adolescents (12–19 years old). Two stool samples obtained from participants were examined with quadruplicate KK for the detection of STH ova according to standard procedures [5]. Subjects were included in the clinical trial if positive for *T. trichiura* on at least one slide on one sample with KK and had a minimum infection intensity of > 24 eggs per gram.

A subsample of one of the two samples of stool (1 g) was preserved in 96% ethanol at room temperature (20–25°) and shipped to Swiss TPH in Basel for PCR analyses. The DNA extraction was performed using the QIAamp DNA Mini kit (Qiagen; Hilden, Germany) with slight modifications from the standard protocol [7]. In brief, samples were washed, centrifuged and separated from ethanol. Approximately 100 mg were transferred into a tube containing garnet beads, suspended in 250 μL of PBS containing 2% polyvinylpolypyrrolidone (pvpp) [35] and frozen at -20 °C overnight.

On the following day the beating process was conducted for 30 s at 3000 rotations per minute (rpm) using a homogenization instrument (Speedmill, Analytica Jena, Jena, Germany). 225 µl tissue lysis buffer (ATL) with 25 µl Proteinase K were added followed by 2 h incubation at 56 °C. Then 500 μl lysis buffer (AL) was added and the samples incubated again at 70 °C for 10 min. After the second incubation, 800 µl supernatant was transferred in a 96 well plate (QIAamp 96 DNA Blood kit) and processed according to the kit protocol. All samples were analyzed with real-time PCR for detection of STH. Primers (Microsynth AG, Switzerland) and Probes (Eurofin Genomics, Ebersberg, Germany) are summarized in supplementary Table 2 and the concentrations of the real-time PCR using TaqMan GeneExpression MasterMix (ThermoFischer, Switzerland) are presented in supplementary Table 3. The thermoprofile on the 7500 ABI real-time Machine (ThermoFischer) was: 2 min at 50 °C, 10 min at 95 °C followed by 45 cycles of 15 s at 95 °C and 1 min at 58 °C. The specificity of these primers was previously tested on a variety of DNA from stool samples confirmed by light microscopy at the diagnostic center of the Swiss TPH to be infected with: *A. lumbricoides, Blastocystis hominis, Cryptosporidium spp., Cyclospora spp., Dientamoeba fragilis, Encephalitozoon intestinalis, Endolimax nana, Entamoeba coli, E. dispar, E. hartmanni, E. histolytica, E. moshkovskii, E. polecki, Enterocytozoon bieneusi, Giardia lamblia, Hymenolepis nana, Iodamoeba bütschili, Sarcocycstis spp., Schistosoma mansoni*, *Taenia spp., Trichostrongylus spp*. and was found to be 100% specific. On each real-time PCR plate and for each target, we included negative and positive controls with different plasmid concentrations (10^2^, 10^4^, and 10^6^ plasmids/µl) containing an insert with the sequence of the STH real-time PCR product. First round PCR detected: *Trichuris spp, Ascaris spp* and Hookworms + S (*Ancylostoma spp, N. americanus* and *S. stercoralis*). All samples positive for hookworms were tested in a second round PCR for hookworm species specification (*N. americanus, A. duodenale*) or *S. stercoralis*.

### Statistical analysis

From the initial dataset, a randomized list of 320 subjects was generated (80 per treatment arm) for PCR analyses. By selecting 80 subjects per treatment group we could achieve 80 percent power of observing a statistically significant difference (at the 5 percent level) between the cure rates of *T. trichiura* according to the two alternative tests (KK vs. PCR) provided that a) the cure rate according to KK under the respective treatment equals at least 30%, b) the corresponding cure rate according to PCR does not exceed 50% of the one according to KK, and c) the correlation between the two tests equals at least 0.4. With a power of 90 percent, the same comparison in the pooled data would lead to a statistically significant difference provided that the true cure rate according to PCR does not exceed 70% of the one according to KK (e.g., with cure rates of 30% according to KK and of ≤ 21% according to PCR).

Data of the amplification curves were entered in an Excel file and statistical analyses were conducted using Stata 14.0 (Lake Drive College Station, Texas).

The cycle threshold (Ct) value was defined as the number of PCR cycles required for the detection of fluorescence signal of the amplified products to exceed the set threshold value. As a consequence, higher quantities of DNA resulted in lower Ct values and vice versa [36].

Because of possible unspecific amplification and to exclude any cross-contamination from highly positive samples, PCR results were considered as negative if the Ct values were above 40 or if no amplification was detected [37]. Since KK is a quantitative diagnostic method, we compared the copies/µl DNA of the PCR and the EPG  obtained by microscopy. KK results were calculated as the mean of the 4 slides performed.

Sensitivities of KK relative to PCR and of PCR relative to KK were calculated for baseline and follow-up separately and for baseline and follow-up results pooled. Cure rate was calculated as the proportion of participants negative for infection (EPG or DNA copy = 0) at follow-up being positive at baseline. 95%-confidence intervals for pooled sensitivities were computed using a logistic regression model with robust standard errors adjusting for longitudinal correlations of test results within individuals. Logistic regression models were used to compare cure rates between different treatment arms and between the PCR and the KK method. The latter analyses involved robust standard error estimates to adjust for correlations of the two types of test results within individuals. Polytomous logistic regression was used to estimate the probabilities of belonging to specific EPG-categories as a function of the copy number according to PCR. The respective figures were obtained using Stata’s margins syntax.

To assess agreement between copy numbers according to PCR and of EPG according to KK, Spearman rank correlation was used among the tests, which were positive according to both PCR and KK. An alternative assessment was based on weighted Cohen’s Kappa, comparing EPG-categories with ad hoc defined copy number categories matching them in size. The latter were obtained through an order-preserving map from the ordered set of EPG-values onto the ordered set of copy numbers. The degree of agreement was classified according to the following grading: very good (0.8–1), good (0.6–0.8), moderate (0.4–0.6), fair (0.2–0.4) and poor (< 0.2).

For a given copy number, the EPG-category to which the respective sample most likely belongs is given by the highest curve at this point.

## Data Availability

All data generated or analyzed during this study are included in this published article.

## Abbreviations

PCR: Polymerase chain reaction
KK: Kato Katz
STH: Soil-transmitted helminth
